# Stent-induced flow disturbances in the ipsilateral external carotid artery following internal carotid artery stenting: a temporary cause of jaw claudication

**DOI:** 10.1007/s00508-017-1224-9

**Published:** 2017-06-09

**Authors:** Georgiana-Aura Giurgea, Markus Haumer, Irene Mlekusch, Schila Sabeti-Sandor, Petra Dick, Martin Schillinger, Erich Minar, Wolfgang Mlekusch

**Affiliations:** 1 Department of Internal Medicine II, Division of Angiology, Vienna General Hospital, Medical University of Vienna, Währinger Gürtel 18–20, 1090 Vienna, Austria; 2 Karl Landsteiner Institute for Angiology und Cardiology, Moedling, Austria

**Keywords:** Carotid artery, Stenting, Jaw claudication, Deglutition, Stenosis

## Abstract

**Background:**

We hypothesize that stenting of the internal carotid artery can immediately impede blood flow to the external carotid artery by either plaque shift or stent coverage of the ostium, and thereby cause ischemic symptoms like ipsilateral jaw claudication.

**Methods:**

Thirty-three patients with high-grade asymptomatic stenosis of the internal carotid artery who underwent endovascular treatment were examined by ultrasound of the external carotid artery and performed an exercise test by chewing chewing gum synchronously to an electronic metronome for 3 min. Tests were performed before, the day after, and 1 week after the stenting procedure. Claudication time was defined as the timespan until occurrence of pain of the masseter muscle and/or chewing dyssynchrony to the metronome for more than 15 s. Ten patients with an isolated, atherosclerotic stenosis of the external carotid artery served as controls.

**Results:**

A significantly reduced claudication time (in seconds) was recorded in patients who underwent carotid artery stenting compared to baseline values; median 89 (interquartile range, IQR, 57 to 124) vs. median 180 (IQR 153 to 180; *p* < 0.001). By categorization of the flow velocity at the external carotid artery into faster or slower as 200 cm/sec, the effect was even accentuated. Stenting values showed improvement 1 week after but did not return to baseline levels. No respective changes were found in controls.

**Conclusion:**

Stenting of the internal carotid artery lead to ipsilateral flow deterioration at the external carotid artery resulting in temporary jaw claudication. This impairment attenuated over the time and was significantly reduced after 1 week.

## Introduction

Over the last years, a very controversial discussion has developed concerning the advantages and disadvantages of carotid artery stenting [1]. However, the overall stroke and death rate of carotid artery stenting (CAS) is closely comparable to endarterectomy and has been found to be non-inferior to surgical treatment [2, 3]. Complications and recurrent stenosis rates are comparable with those reported after carotid endarterectomy [3, 4].

In the vast majority of patients, the atherosclerotic lesions are located at the carotid bifurcation involving the very proximal segment of the internal carotid artery (ICA). The orifice of the external carotid artery (ECA) is also frequently affected. It has become common practice that the stents are implanted across the bifurcation towards the internal carotid artery by covering the orifice of the external carotid artery.

Ischemic symptoms such as facial pain, jaw claudication, or neck pain have been reported to be associated with high-grade stenosis of the ECA [5, 6].

Furthermore, the stent-based coverage of the orifice of the ECA has been shown to accelerate the atherosclerotic narrowing at the ECA [7].

It is currently unknown whether plaque shift from the internal carotid artery and/or the implantation of a stent over the orifice of the ECA during the course of CAS can cause acute ischemic symptoms in the vascular territory of the ECA.

We hypothesize that CAS decreases blood supply in the ipsilateral territory of the ECA and causes clinical ischemic symptoms like jaw claudication.

## Methods

The study was designed as a prospective cohort study. The study complies with the Declaration of Helsinki and was approved by the Ethics committee of the Medical University of Vienna. Written informed consent was obtained from all patients and controls.

### Patients

#### Inclusion and exclusion criteria

Inclusion criteria were symptomatic atherosclerotic obstructive lesions in the extracranial ICA scheduled for CAS. The exclusion criterion was inability to chew a chewing gum.

### Exercise test

The patients were instructed to chew a sugarless chewing gum (Orbit, Wrigley Company, Chicago, IL, USA) synchronously to an electronic metronome at 80 beats per minute for 3 min. The rational of choosing a pace of 80 beats per minute is based on the idea of challenge at the maximal chewing capacity, as it might be more likely to be impaired as it is at 15 times per minute. The exercise test was performed the day before, the day after the stenting procedure, and 1 week thereafter.

Patients were instructed to report the occurrence of any complaints in the ipsilateral mastication muscles during the chewing exercise immediately. In case of chewing out of rhythm, the patient was advised to return to the predefined rhythm. If the patient remained out of rhythm, e. g., below 80 chewing cycles per minute, for ≥15 s, this was also classified as a symptom of arterial insufficiency. Claudication time was defined as the timespan to the occurrence of complaints or the development of an ongoing chewing dyssynchrony with the metronome. Patients were classified as normal when they completed the test without symptoms throughout 3 min.

### Controls

Patients with significant stenosis of external carotid artery served as a control group and an internal validation of our chewing test. They underwent exercise testing at baseline, 1 day later, and 1 week thereafter.

### Color-coded duplex sonography

Color-coded duplex sonography examinations were performed on an Acuson Sequoia M512 platform equipped with a 5-MHz linear array probe (Siemens, Erlangen, Germany) by a single experienced vascular technologist (IM).

Examinations were performed prior to the procedure (baseline) and at day one after the stent placement. Control patients underwent just the baseline duplex scanning.

Peak systolic velocities (cm/sec) of the external carotid artery were recorded right distal to the overstented orifice of the ECA and entered into final analysis.

### Statistical methods

Continuous data were presented as the median and the interquartile range (IQR; range from the 25^th^ to the 75^th^ percentile). Percentages were calculated for dichotomous variables. The chi-square test was used for comparison of categorical variables. Continuous variables were compared by means of the Mann–Whitney U test. Related samples were compared by Kruskal–Wallis testing and McNemar analysis. All *p* -values are calculated two sided; a *p* -value < 0.05 is considered as statistically significant. Calculations are performed with SPSS for Mac (Version 18.0, SPSS Inc., Chicago, IL, USA).

## Results

**Fig. 1:**
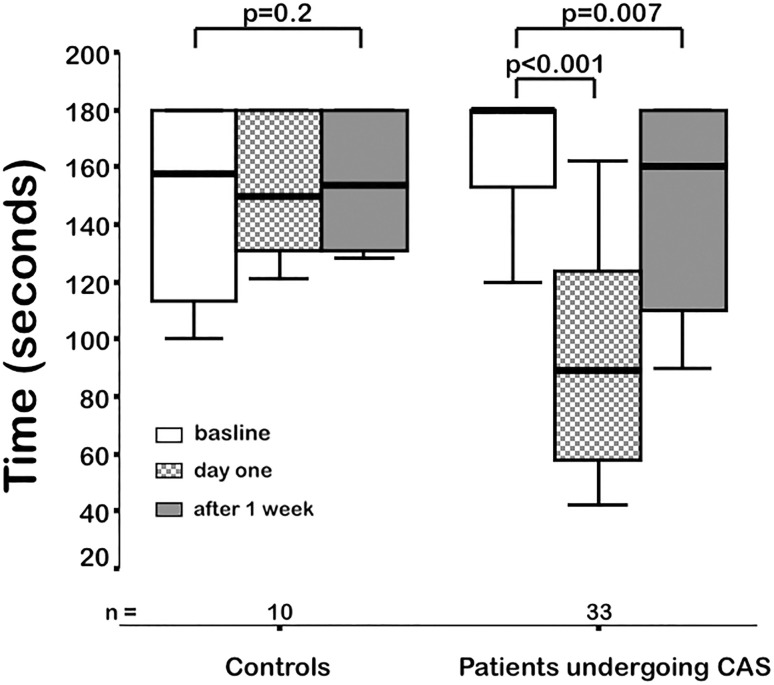
Exercise duration until claudication in the masseter muscle occurs in patients and controls. *CAS* carotid artery stenting

We studied 33 patients who were scheduled for elective carotid artery stenting due to high-grade stenosis of the ICA and 10 controls with significant stenosis of the external carotid artery. Demographics are given in Table 1. At baseline, peak systolic flow velocities of the ECA were significantly higher in controls. The claudication time at baseline did not vary significantly but it was significantly shorter in patients 1 day after CAS (Table 2; Fig. 1). However, the timespan during the exercise test increased significantly 1 week after stent implantation, although it failed to reach baseline levels (Fig. 1).

**Table 1:** Demographic variables

	Patients undergoing CAS *N* = 33	Control *N* = 10	*P*-value
*Female sex*	6 (18%)	5 (50%)	0.04
*Age (years)*	61 (58 to 65)	66 (56 to 82)	0.17
*Current smoker*	12 (36%)	2 (20%)	0.6
*Dyslipidemia*	32 (97%)	10 (100%)	0.58
*Art. hypertension*	29 (88%)	10 (100%)	0.25
*Diabetes mellitus*	14 (42%)	3 (30%)	0.86
*Known PAOD*	16 (48%)	4 (60%)	0.27
*Known CAD*	16 (48%)	3 (30%)	0.62
*Statin use*	32 (97%)	10 (100%)	0.58

*CAS* carotid artery stenting, *PAOD* peripheral artery occlusive disease, *CAD* coronary artery disease, *Art. *arterial

**Table 2:** Variables on hemodynamics of the external carotid artery and details of the respective chewing time

	Patients undergoing CAS *N* = 33	Control *N* = 10	*P*-value
External carotid artery PSV baseline (cm/s)	83 (80 to 152)	190 (170 to 220)	<0.001
External carotid artery PSV day one (cm/s)	230 (155 to 260)	190 (170 to 220)	0.4
Claudication time baseline (s)	180 (153 to 180)	157 (110 to 180)	0.23
Claudication time day one (s)	89 (57 to 124)	149 (131 to 180)	<0.001
Claudication time after 1 week (s)	160 (108 to 180)	154 (131 to 180)	0.5

*PSV* peak systolic velocity, *CAS* carotid artery stenting

**Fig. 2:**
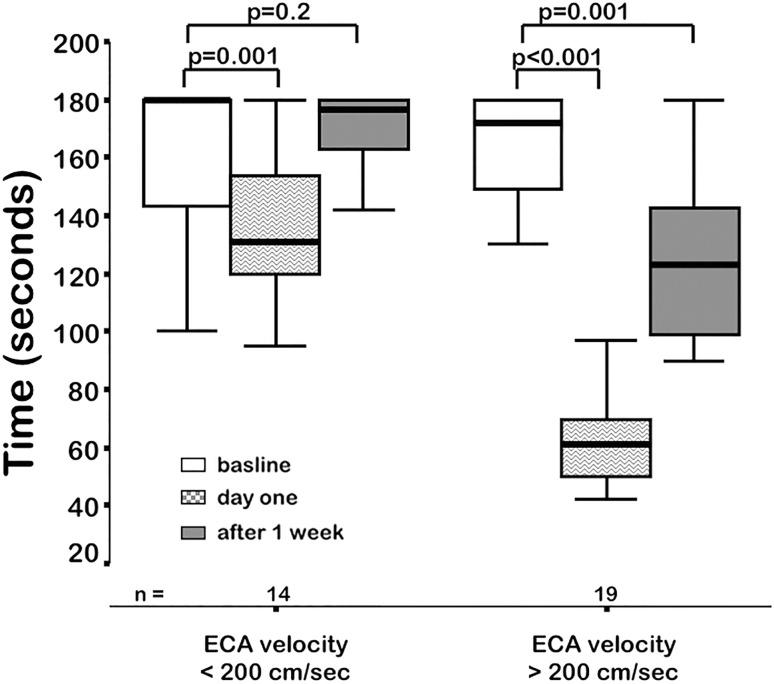
Exercise duration until claudication in the masseter muscle occurs in patients with respect to the maximum flow velocities in the external carotid artery. *ECA* external carotid artery

The peak systolic velocities of the external carotid artery were directly correlated with the occurrence rate and time course of jaw claudication. Claudication time was shorter and improved less over the time course of 1 week in patients with flow velocities ≥ 200 cm/s as compared to patients with flow velocities < 200 cm/s (Fig. 2).

## Discussion

As far as we know, no other study has yet analyzed the impact of CAS on clinical symptoms related to an acute iatrogenic narrowing of the ipsilateral ECA by either overstenting of the orifice and/or plaque shift. The lack of scientific proof does not necessarily mirror a reduced clinical awareness, as a few case reports describe ischemic symptoms due to ECA narrowing [8, 9]. It is known that the ECA frequently develops significant narrowing over the time after CAS [10]. However, stenosis of the ECA per se has been regarded as irrelevant for the overall clinical course [7] or the rate of ECA occlusion [11].

We demonstrated that CAS has an acute negative effect on the hemodynamics, as determined by a significant increase of the peak systolic velocity at the origin of the ECA. Consistently, a considerable number of patients who underwent CAS developed jaw claudication in a standardized chewing exercise test. This functional impairment was directly linked to the level of blood flow alteration and it improved significantly within 1 week. The development of collateral pathways is certainly the most plausible explanation for this improvement.

For affected patients, such ischemic symptoms could temporarily affect their subjective wellbeing. Especially, patients with pre-existing atherosclerotic stenosis of the contralateral ECA are at risk of severe jaw claudication after deploying stents across the orifice of the ECA. These patients should be directly informed that accentuated flow disturbances of the ECA due to the stent procedure could result in symptoms, which are, in most of the cases, not severe enough to be clinically important.

### Limitations

The presented study was small-numbered. However, the shown effect was of clear significance, which could not be expected to be bigger in a larger patient series. An extended observation time should reveal whether iatrogenic jaw claudication will subside completely. Moreover, the general clinical impact is, due to the transient nature, rather mild. Transient jaw claudication as side effect of carotid stenting is compared to the usual complications of the surgical approach neglectable and just of scientific interest. Finally, due to the transient nature, we did not systematically evaluate the impact of the phenomenon of jaw claudication on the quality of life of our patients.

## Conclusion

Flow alteration in the ECA after CAS is associated with a significantly reduced chewing competence in standardized exercise testing due to jaw claudication. The intensity and duration of this impairment were linked to the level of flow acceleration. The impairment attenuated over the time and was significantly reduced 1 week later.
